# *MAEL* gene contributes to bovine testicular development through the m5C-mediated splicing

**DOI:** 10.1016/j.isci.2023.105941

**Published:** 2023-01-06

**Authors:** Shenhe Liu, Xiaoya Ma, Zichen Wang, Feng Lin, Ming Li, Yali Li, Liu Yang, Hossam E. Rushdi, Hasan Riaz, Tengyun Gao, Liguo Yang, Tong Fu, Tingxian Deng

**Affiliations:** 1 College of Animal Science and Technology, Henan Agricultural University, Zhengzhou 450046, China; 2 Guangxi Provincial Key Laboratory of Buffalo Genetics, Breeding and Reproduction Technology, Buffalo Research Institute, Chinese Academy of Agricultural Sciences, Nanning 530001, China; 3 Wuhan Benagen Technology Co, Ltd, Wuhan 430000, China; 4 Department of Animal Production, Faculty of Agriculture, Cairo University, Giza 12613, Egypt; 5 Department of Biosciences, COMSATS University Islamabad, Sahiwal Campus, Punjab, Pakistan; 6 China Ministry of Education, Key Laboratory of Agricultural Animal Genetics, Breeding and Reproduction, College of Animal Science and Technology, Huazhong Agricultural University, Wuhan, China

**Keywords:** Animals, Developmental genetics, Transcriptomics

## Abstract

Knowledge of RNA molecules regulating testicular development and spermatogenesis in bulls is essential for elite bull selection and an ideal breeding program. Herein, we performed direct RNA sequencing (DRS) to explore the functional characterization of RNA molecules produced in the testicles of 9 healthy Simmental bulls at three testicular development stages (prepuberty, puberty, and postpuberty). We identified 5,043 differentially expressed genes associated with testicular weight. These genes exhibited more alternative splicing at sexual maturity, particularly alternative 3’ (A3) and 5’ (A5) splice sites usage and exon skipping (SE). The expression of hub genes in testicular developmental stages was also affected by both m6A and m5C RNA modifications. We found m5C-mediated splicing events significantly (p < 0.05) increased *MAEL* gene expression at the isoform level, likely promoting spermatogenesis. Our findings highlight the complexity of RNA processing and expression as well as the regulation of transcripts involved in testicular development and spermatogenesis.

## Highlights

• Transcriptome complexity of the bull testis has been characterized
• Hub genes related to testicular development and spermatogenesis are identified
• RNA processing events are shown to regulate distinct expression of hub genes
• The m5C-mediated splicing events significantly increased expression of *MAEL* isoforms

## Graphical abstract

**Figure:**
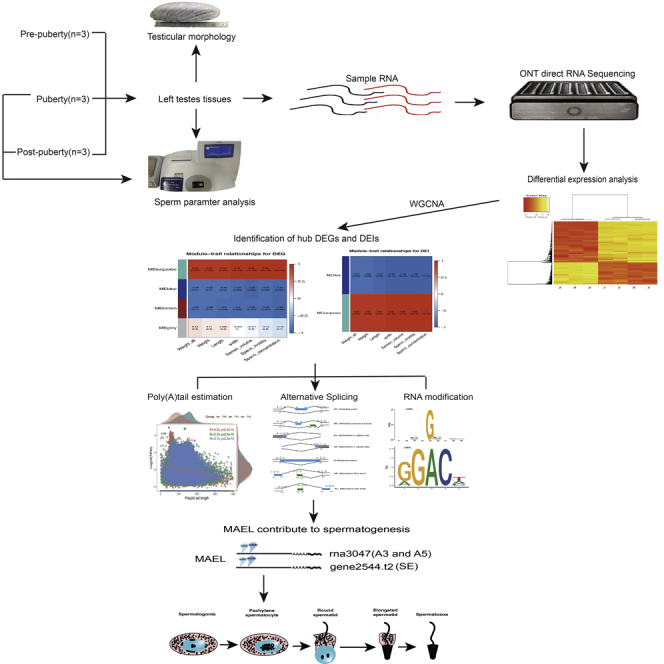

## Introduction

Simmental cattle are renowned worldwide for their rapid growth potential, meat quality, and environmental adaptability. During the last two decades, Simmental breed has been used for crossbreeding with some Chinese indigenous beef breeds to improve their production efficiency.^1^^,^^2^^,^^3^ The primary reproductive organ in males (testis) plays a fundamental role in spermatogenesis and steroidogenesis. The linear increase in size of the testicles along with proportional growth in cellular compartments is pivotal for the establishment of spermatogenesis, early maturity, and selection of the potential breeding sires.^4^^,^^5^^,^^6^ Farm animals vary in the age at sexual puberty and maturity. In this regard, Lunstra et al.^7^ reported that puberty is usually attained at 42 weeks but varies from 38 to 46 weeks, depending on the breed of cattle. Neto et al.^8^ found that the age at puberty in Simmental bulls was 13.42 ± 3.02 months and their sexual maturity was achieved at 21.43 ± 6.6 months. In addition, sexually mature bulls had utmost testicular size along with normal sexual behavior and over 70% progressive sperm motility compared with immature bulls.^9^ A good understanding of the prepubertal and postpubertal development of the bull testis would provide a strong basis for an ideal progeny testing program in a young bull’s life before securing his ranking as a semen donor.

Testicular development is predominantly characterized by the development and differentiation of germ cells (GCs) and sertoli cells (SCs).^10^^,^^11^ SCs are essential for spermatogenesis not only through direct interaction with GCs within the seminiferous epithelium but also by forming the blood-testis barrier (BTB) to provide morphological, nutritional, and immune support for GC development.^12^ Notably, spermatogenesis is strictly regulated by the expression of stage-specific genes at both transcription and post-transcription levels.^13^ For instance, DEAD-box polypeptide 4 (DDX4) gene expressed in the human, pig, and horse germ cells can promote cell proliferation.^14^^,^^15^^,^^16^ The maelstrom spermatogenic transposon silencer (MAEL) localized in satellite body (SB) of pachytene spermatocytes at stage IX–X and in chromatid body (CB) in round spermatids was colocalized with DDX4, DDX25, and MIWI in both nuage and non-nuage structures.^17^ FK506-binding protein 6 (FKBP6), also known as FKBP36, plays a crucial role in the sex-specific fertility and regulation of homologous chromosome pairing in mammalian meiosis.^18^^,^^19^^,^^20^

Spermatogenesis is a complex and dynamic process controlled by genetic and epigenetic factors. Determining how these genes are regulated by epi-transcriptomic regulators (e.g., prevalent RNA modifications like N6-methyladenosine [m6A] and 5-methylcytosine [m5C]) is highly important for clarifying bovine testicular development and spermatogenesis. Alternative pre-mRNA splicing plays important roles in co-transcriptional and post-transcriptional regulation of gene expression during spermatogenesis. Studies focusing on alternative splicing (AS) on spermatogenesis supported the notion that the development of testis is regulated by a higher level of AS than other tissues.^21^ Both m6A and m5C are the most abundant modifications in mRNAs and play a pivotal role in regulating AS and RNA degradation.^22^^,^^23^ Poly(A) tails are also very important for RNA processing. The length of the poly(A) tail is correlated with mRNA translation efficiency and transcript stability to regulating mRNA.^24^^,^^25^ However, information on the molecular function of these transient stages of testicular development and spermatogenesis is still limited. Increasing our understanding of the bovine testicular development and spermatogenesis is of great importance for the evaluation of promising bulls.

Among the long-read sequencing methods, direct RNA sequencing (DRS) is a powerful and effective tool for delineating the dynamics and complexity of transcriptome.^26^ DRS plays a vital role in dissecting the genotype-phenotype relationships that could reflect all aspects of testicular growth and development. To date, DRS technology has been adopted in identifying full-length splice isoforms,^27^ exploring post-transcriptional events,^28^ characterizing poly(A) tail lengths of transcripts,^29^ improving the existing genome assembly,^30^ and detecting RNA modifications.^31^ Nevertheless, the DRS technology adopted for clarifying the transcriptome complexity has still not been widely applied in farm animal species, which delays the characterization and application of true transcriptomics in livestock industry.

In this regard, the objective of the present study was to explore the dynamics and complexity of testicular transcriptome in Simmental bulls at three different developmental stages using the DRS technology. We first aimed to generate an atlas of the testis transcriptome of the bulls. Subsequently, we aimed at identifying the hub genes related to testicular development and spermatogenesis at various developmental stages. Furthermore, we characterized the functional roles of RNA processing events in hub genes related to testicular weight of Simmental bulls.

## Results

### Testis source description

Previous studies have demonstrated a positive correlation between testicular development and semen quality.^32^^,^^33^^,^^34^ Hence, an examination of testicular morphology and semen quality parameters is crucial to understanding testicular development-semen quality relationships. Herein, we described the testis’ morphological features (testicular weight, TM; testicular length, TL; and testicular width, TW) in 9 Simmental bulls at three developmental stages of the testis: prepuberty (TY0, n = 3), puberty (TY1, n = 3), and postpuberty (TY2, n = 3). The results showed significant (p < 0.05) differences in testicular phenotypic parameters among the different developmental stages (Table 1). Notably, the values of TM differed significantly (p < 0.05) among the study groups. Moreover, semen quality parameters such as semen volume (SV), sperm motility (SM), and concentration (CO) were significantly (p < 0.05) higher in TY2 compared to TY1. Pairwise correlation coefficients among age, testicular measurements, and semen quality traits indicated strong correlations (Figure S1), indicating to their role in sperm production. The histomorphology study of bull testis revealed significant (p < 0.05) differences between prepuberty and postpuberty stages. The diameter of seminiferous tubules increased gradually with the testicular development when observed at 10× microscopic magnification. This examination demonstrated that the testes at TY2 had the longest diameter of seminiferous tubules (Figure S2E), followed by the TY1 (Figure S2C) and TY0 (Figure S2A). At 40X, we observed that TY1 (Figure S2D) and TY2 (Figure S2F) had more advanced developmental stages of spermatozoa than TY0 (Figure S2B).

**Table 1:** Statistics for testicular measurements and semen quality traits in Simmental bull testes at three developmental ages (Mean ± SEM)

Variable	TY0 (n = 3)	TY1 (n = 3)	TY2 (n = 3)
Age, months	0.10 ± 0.00	13.28 ± 0.31	22.39 ± 0.20
TM, g	5.11 ± 0.63^c^	172.40 ± 3.34^b^	323.48 ± 11.70^a^
TL, cm	4.17 ± 0.38^c^	10.17 ± 0.57^b^	13.50 ± 0.21^a^
TW cm	1.80 ± 0.12^c^	5.77 ± 0.07^b^	7.43 ± 0.13^a^
SV, mL	0.00 ± 0.00	5.83 ± 0.07^b^	7.34 ± 0.13^a^
SM, %	0.00 ± 0.00	0.61 ± 0.004^b^	0.67 ± 0.002^a^
CO, ×10^9^/mL	0.00 ± 0.00	1.01 ± 0.53^b^	1.21 ± 0.56^a^

^a,b, and c^The different superscripts in the same row show significant differences (p < 0.05). TY0 = prepuberty; TY1 = puberty; TY2 = post puberty; SV = Semen volume; SM = Sperm motility; CO = Concentration; TM = Testicular weight; TL = Testicular length; TW = Testicular width.

### Statistics of nanopore DRS data

**Figure 1:**
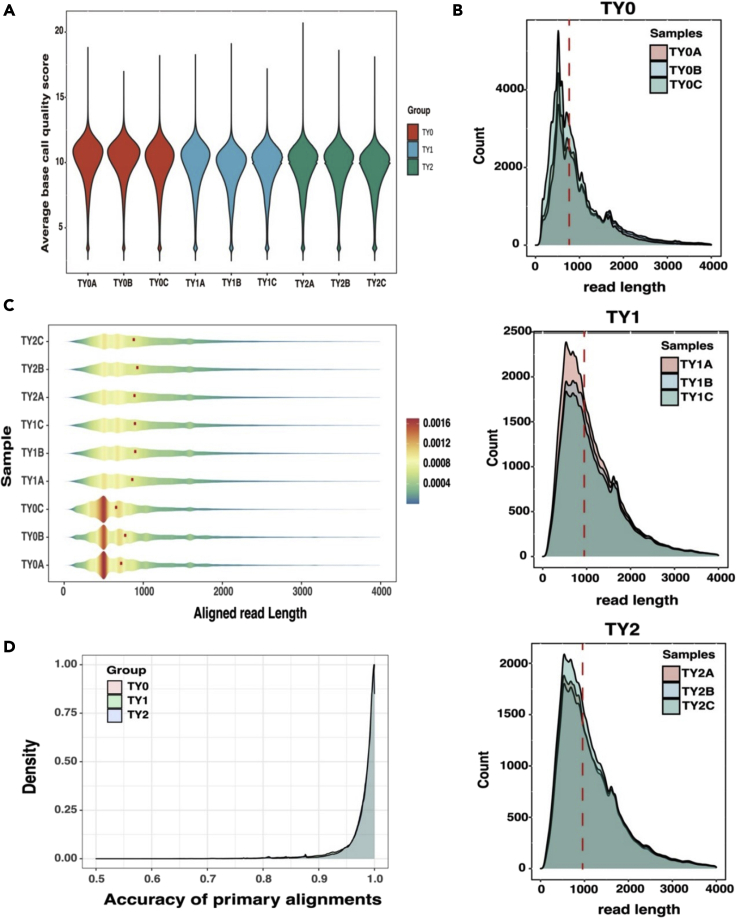
Summary statistics of Oxford Nanopore Technologies (ONT) data (A) Violin plot displays the distribution of quality scores across all samples. (B) Density plot indicates the distributions of read count over read length among groups. The red dashed represents the median read length for each sample. (C) Violin plot displays the distribution of aligned read length across all samples. The red point represents the median read length for each sample. (D) Density plot indicates the distributions of the accuracy of primary alignments among the studied groups.

In total, 50.77 million long reads were generated (Table 2). For each stage, we generated at least 4.0 million reads with an average read length of 1,663.00, 1,381.50, and 1,382.83 bp for TY0, TY1, and TY2, respectively. After filtering, about 6.0, 5.0, and 4.6 million clean reads for TY0, TY1, and TY2 were obtained, respectively. These reads had a median quality score of 10 across all samples (Figure 1A), providing high-quality DRS data for subsequent analysis.

**Table 2:** Summary of Nanopore native RNA read statistics for bovine testis transcriptomes at three developmental stages

Read characteristics	TY0			TY1			TY2		
	TY0A	TY0B	TY0C	TY1A	TY1B	TY1C	TY2A	TY2B	TY2C
Total reads	6,338,070	5,854,672	7,533,763	5,809,796	5,276,222	4,829,863	4,855,136	4,857,834	5,418,378
Average length	1,720	1,835	1,434	1,347	1,391	1,406.50	1,390	1,410.50	1,348
N50 length	2,732	2,905	2,195	2,160	2,252	2,237	2,230	2,272	2,158
Maximum read length	14,464	14,228	11,183	12,719	15,411	12,365	15,580	15,792	14,431
Clean reads	5,979,661	5,573,264	6,990,324	5,342,331	4,729,269	4,3956,30	4,407,301	4,428,762	4,853,541
Mapped reads	5,915,918	5,534,295	6,906,174	5,302,284	4,685,889	4,358,840	4,365,705	4,394,502	4,812,794
Reads aligned to genome (%)	98.93	99.3	98.8	99.25	99.08	99.16	99.06	99.23	99.16
Reads aligned to transcriptome (%)	89.45	90.27	87.38	87.39	86.14	86.52	87.21	88.28	87.69

Furthermore, we observed that the DRS data had high genome mappability, with an average of 99.11% across all samples (Table 2). Consistent with this, when the long reads were mapped against the existing bovine transcriptome (*Btau_5.0.1*), the overall mappability dropped to 87.81%. These results made it possible to anticipate that a large number of novel transcripts will probably appear in the bovine transcriptome. The distribution of read count over read length among all samples is present in Figure 1B. Reads had a median length of 766, 946, and 958 nucleotides for TY0, TY1, and TY2, respectively. The number of short reads in TY0 group was higher than that of the other groups. The overall aligned median length was approximately 834 nucleotides across all samples (Figure 1C). Among the groups, TY2 had the highest median-aligned read length (899 nucleotides), followed by TY1 (886 nucleotides) and TY0 (717 nucleotides). In addition, most primary alignments had more than 98% of median accuracy among the studied groups (Figure 1D). The higher mapping rate and longer aligned read length obtained in the present study enhance the expectation that almost all reads were assigned to a single gene and transcript isoform.

### Nanopore DRS reveals characteristics of bovine testicular transcriptome

**Figure 2:**
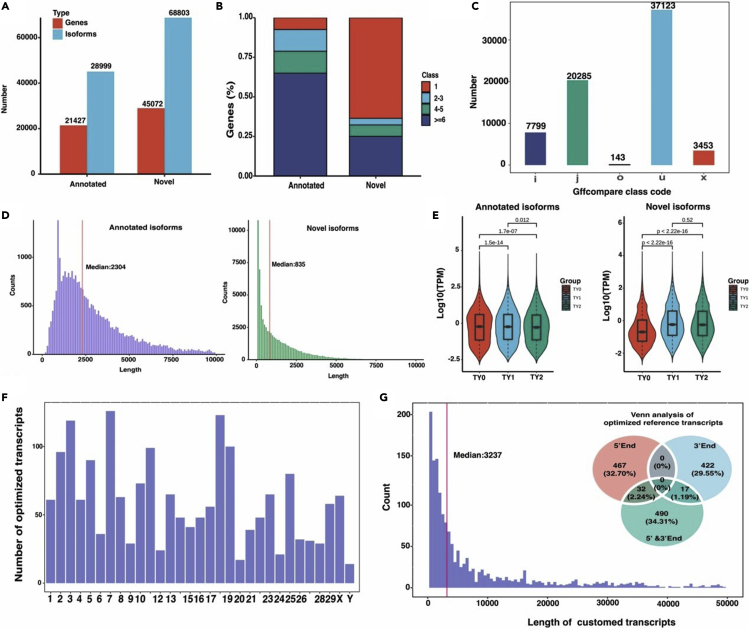
Identification of transcriptome complexity for bovine testis (A) Barplot revealed the numbers of the annotated and novel isoforms and genes. (B) Distribution of isoform among NCBI annotated genes and novel genes. (C) Number of novel transcripts based on gffcompare class code. (D) Distribution of isoform length among NCBI annotated genes and novel genes. (E) Expression patterns of known and novel transcripts across different groups. (F) Chromosome distribution of optimized transcripts. (G) Histogram plot of length of optimized transcripts for the different genomic regions and Venn analysis of optimized reference transcripts.

To characterize the transcriptome complexity of bovine testis, we first assembled the transcript sequences using the DRS data. A total of 97,802 collapsed transcript sequences encoding 56,560 genes were generated, consisting of 28,999 annotated and 68,803 novel isoforms (Figure 2A). Surprisingly, 45,072 novel genes were identified, which were over 2-fold of the annotated genes (21,427). Over 60% of novel genes were mono-exon compared to the NCBI annotated genes where approximately 60% of the genes were multi-exon (Figure 2B). In comparison to the reference annotation, the majority of the novel transcripts were annotated to the gffcompare class u (no overlap, n = 37,123), followed by the class j (contains a reference gene within intron, n = 20,285), i (involved in reference intron, n = 7,799), x (opposite strand, n = 3,453), and o (exon overlaps with reference transcript, n = 143) (Figure 2C). Moreover, the distribution of the annotated and novel transcripts indicated that the median lengths of all unique annotated and novel transcripts were 2,304 and 836 bp, respectively (Figure 2D).

To explore the expression patterns of the annotated and novel transcripts, we performed expression analysis across all groups (Figure 2E). The results revealed that the expression level of the annotated isoforms was comparable to the novel isoforms. All the identified transcripts except for the novel transcript between TY1 and TY2 displayed significant (p < 0.05) expression differences among the studied groups. These findings point out that the differentially expressed transcripts might appear more before and after sexual maturity.

For the bovine reference transcripts from the NCBI annotation, we performed the structure optimization of the transcript based on the collapsed gene transfer format (GTF) file. The results showed that a total of 1,861 reference transcripts had been structurally optimized (Table S2), and the majority of these optimized transcripts occurred on chromosomes 18, 7, and 3 (Figure 2F). Notably, a total of 467 and 422 optimized positions of unique reference transcripts were located at the 5′ and3′ ends, respectively, while 490 occurred at both 5′ and3′ ends (Figure 2G). In addition, we observed that the median length difference between reference and optimized transcripts was 3,237 bp (Figure 2G), demonstrating that the actual length of the Coding DNA Sequence (CDS) regions is longer than the original bovine annotation file (***Btau_5.0.1***).

### Large difference between DEGs and DEIs during testicular development

**Figure 3:**
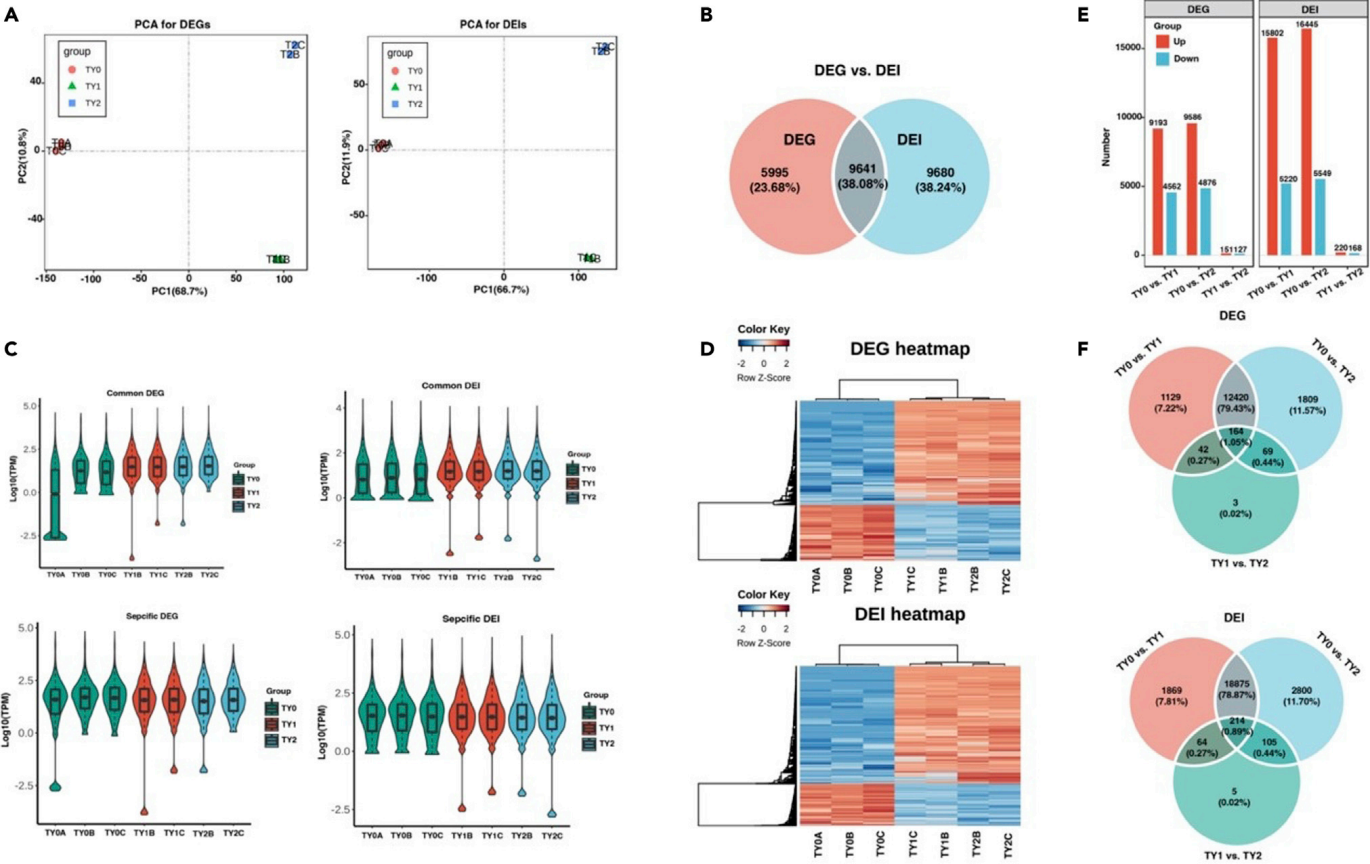
Identification of DEGs and DEIs across testes samples (A) PCA analysis of DEGs and DEIs. (B) Venn analysis of DEGs and DEIs. (C) Expression distribution of the shared and specific DEGs and DEIs. (D) Heatmap of DEGs and DEIs. (E) Numbers of up- and downregulated DEGs and DEIs. (F) Venn analysis of DEGs and DEIs between pairwise comparison groups.

To evaluate stage-specific expression at the gene and isoform levels, we examined the differential expression genes (DEGs) and differential expression isoforms (DEIs) between pairwise comparison groups using DESeq2. Prior to the differential expression analysis, we also performed principal-component analysis (PCA) for all samples. The PCA showed that two samples (TY1A and TY2A) exhibited poor biological reproducibility (Figure S3). Consequently, the two samples were excluded from further analysis. The PCA of the DEGs and DEIs at both gene and isoform levels indicated that the remaining 7 samples were consistent with their respective groups based on their biological characteristics (Figure 3A). A total of 15,636 DEGs and 23,932 DEIs were determined between the pairwise comparison groups, of which 9,641 genes were shared between DEGs and DEIs (Figure 3B), suggesting a moderate overlap between the genes with a stage-specific expression at the gene and isoform levels. These DEGs and DEIs had relatively consistent expression trends between TY1 and TY2 groups, but their expression differences were significantly (p < 0.05) different than that of TY0 group (Figure 3C). This finding was later supported by the heatmap analysis (Figure 3D).

For the DEGs, we observed that the TY1 and TY2 groups respectively had 4,562 and 4,876 downregulated genes compared to the TY0 group; correspondingly, 9,193 and 9,585 upregulated genes were detected (Figure 3E). As for the DEIs, a total of 5,220 and 5,549 downregulated isoforms were identified in the TY1 and TY2 groups, respectively, compared to the TY0 group; correspondingly, 15,802 and 16,445 upregulated isoforms were discovered. In comparison with the TY1, the TY2 group had 127 downregulated and 151 upregulated DEGs and 168 downregulated and 220 upregulated DEIs.

Furthermore, 164 DEGs (1.05% of total DEGs) and 214 DEIs (0.89% of total DEIs) were shared across the pairwise comparison groups, implying that a few stage-specific genes were shared during the testicular development of bulls (Figure 3F). Despite this finding, we observed that a total of 12,420 DEGs and 18,875 DEIs were shared between TY0-TY1 and TY0-TY2. The shared DEGs and DEIs accounted for 79.43% and 78.87% of the total DEGs and DEIs, respectively. As mentioned above, TY0 belongs to the stage before sexual maturity, while TY1 and TY2 represent the stages around sexual maturity, since the animals included in the last two groups can produce sperms. Thus, it can be inferred from these results that most DEGs and DEIs are involved in testicular development and spermatogenesis.

### Effect of RNA processing events on hub genes related to testis weight

Considering that the majority of DEGs and DEIs were involved in testicular development and spermatogenesis, we evaluated their co-expression relationships using the Weighted Gene Co-Expression Network Analysis (WGCNA) algorithm, aiming to define trends in gene co-expression across the testicular tissues at different developmental stages. A total of 3 and 2 co-expression modules were detected in the DEGs and DEIs datasets, respectively (Figure S4). Notably, most DEGs (n = 12,529) and DEIs (n = 19,123) were assigned to the turquoise module. Interestingly, all the identified modules of DEGs and DEIs were significantly (p < 0.01) associated with the testicular morphology and semen phenotypic traits. For them, both DEGs and DEIs within the turquoise module had strong positive correlations with the testicular morphology and semen phenotypic traits, whereas the other modules displayed negative correlations.

**Figure 4:**
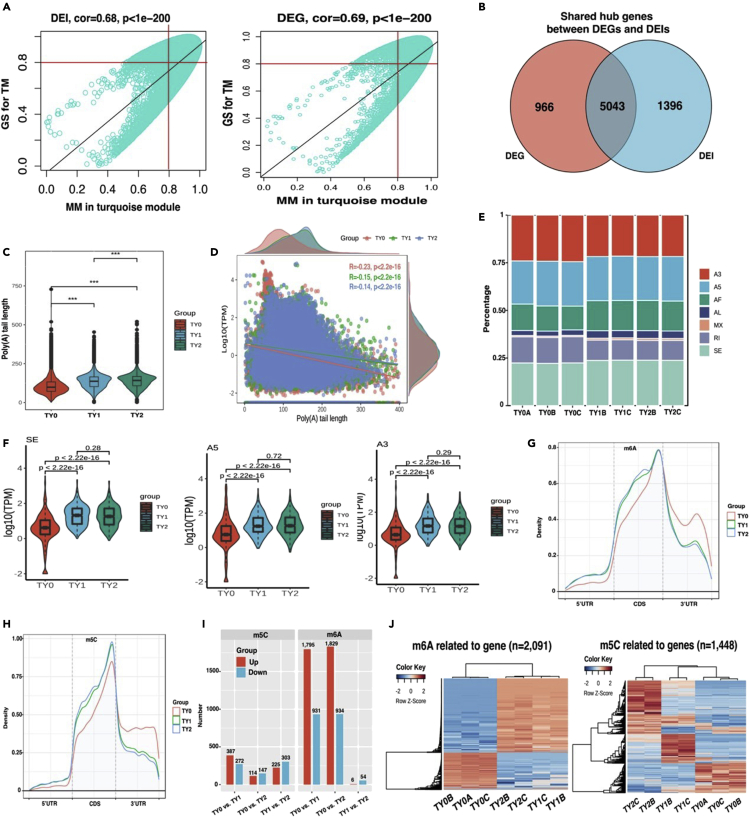
WGCNA, poly(A) tails, alternative splicing, and RNA modifications analysis reveal the hub genes related to testis weight (A) Scatterplot of module eigengenes in the turquoise module from DEGs and DEIs based on WGCNA analysis. (B) Venn diagram of the DEGs and DEIs hub genes. (C) Distribution of poly(A) tail length across different groups. (D) Scattered Marginal Plot display the relationships between expression and poly(A) tail length across different groups. (E) Percentage distribution of AS events among all the samples. (F) Violin plot displays the relationships between expression level and AS types across different groups. (G) Metagene plots displaying the regions of m6A peaks identified across the transcripts in TY0, TY1, and TY2 groups. (H) Metagene plots displaying the regions of m5C peaks identified across the transcripts in TY0, TY1, and TY2 groups. (I) Barplot displaying the number of up- and down-regulated DEIs related to m5C or m6A between comparison groups. (J) Heatmap plot of m6A and m5C related to hub genes among the studied groups.

As the testis weight is an important indicator for measuring testicular development and sperm production, we further identified the hub genes in the turquoise module that were associated with TM. In our previous study,^35^ the hub genes were selected according to the following threshold levels: (Association between gene expression and each module eigengene) MM > 0.8, (Correlation between gene expression and each trait) GS > 0.8, and p value <0.05. In the present study, a total of 8,607 DEGs and 11,639 DEIs highly related to TM in the turquoise module were identified and defined as the putative hub genes (Figure 4A). Venn’s analysis further revealed that 5,043 shared genes between DEGs and DEIs were identified and well defined as the real hub genes (Figure 4B). To validate the reliability of this result, qPCR analysis was conducted and showed that a similar tendency was observed between the mRNA expression level from DRS and qPCR (Figure S5).

Poly(A) tails are known as critical regulators of translation and RNA stability.^25^ In specific, poly(A) length distributions are dynamic in the developing *Drosophila melanogaster* oocyte and embryo.^36^ To determine if there were comparable shifts in the length distribution of poly(A) tails in the well-defined 5,043 hub genes, we examined poly(A) tail lengths across the developmental stages in bull testis. An unexpected observation was that the length of poly(A) tail generated significantly (p < 0.001) different changes among the three stages (Figure 4C). Meanwhile, the poly(A) tail length had a significant negative correlation with the transcripts per million (TPM) values of hub genes among all groups (p < 0.001; Figure 4D). However, this correlation explained only a small fraction of the overall variation in the data, with the maximum R value of 0.23. During the sexual maturity stages (TY1 and TY2 groups), the slopes of the regression lines between the poly(A) tail length and expression level were much shallower, and the corresponding R values were weaker, with R values ranging from 0.14 to 0.15. Overall, these findings demonstrate that the inverse relationship between poly(A) length and expression level may vary depending on the developmental stage of the testis.

AS is known to increase the complexity of gene expression and plays an important role in cellular differentiation and organism development.^37^ To determine the relationship between the AS and the expression of hub genes, the corresponding AS patterns in bull testis at different developmental stages were examined in the present study. We identified 12,792 AS events in the hub genes, including the alternative 5′ splice site (A5), alternative 3′ splice site (A3), alternative first exon (AF), alternative last exon (AL), mutually exclusive exons (MX), retention intron (RI), and skipping exon (SE), and the intersection of AS events between the studied groups (data not shown). The most abundant splicing events were the SE (23.63%), followed by A5 (23.16%), A3 (21.83% s), AF (15.71%), RI (10.79%), AL (3.92%), and MX (0.98%) (Figure 4E). To further explore the effects of differential AS events on the expression levels of hub genes, the analysis of differential expressed AS (DAS) events between the pairwise comparison groups was carried out. A total of 2,795 DAS events were detected, of which 655 belonged to the A3 type, followed by A5 type (653), SE type (552), AF type (504), RI type (357), AL type (71), and MX type (3) (Figure S6). Interestingly, different DAS events displayed significant expression differences between TY0 and TY1/TY2 groups, but this scenario was not found between TY1 and TY2 (Figure 4F; S7). Such results indicate that the hub genes could regulate the expression level through different AS events, thereby influencing the testis weight.

Both m6A and m5C are the most common internal modifications in mRNAs involved in various RNA metabolism and regulations.^38^ To investigate whether the modifications in the expression of hub genes were contributed by m6A or m5C, the ratio of m6A and m5C was calculated and quantified. The results showed that 32,716 m6A (Table S3) and 209,597 m5C sites (Table S4) were identified, corresponding to 3,549 and 4,128 hub genes, respectively. The average number of m6A and m5C modification sites for each transcript was 4.12 and 25.58, respectively. Their distribution revealed that both sites were enriched near the stop codon and 3′UTR regions (Figures 4G and 4H). To validate the reliability of m6A sites based on DRS, we performed a MeRIP-Seq analysis using our previous Xia-Nan testis data.^39^ The distribution of m6A peaks confirmed the enrichment near the stop codon and 3′UTR region (Figure S8A), which is similar to the DRS results. These findings imply that the identified m6A and m5C modification sites are highly reliable and could be used for further analyses.

**Figure 5:**
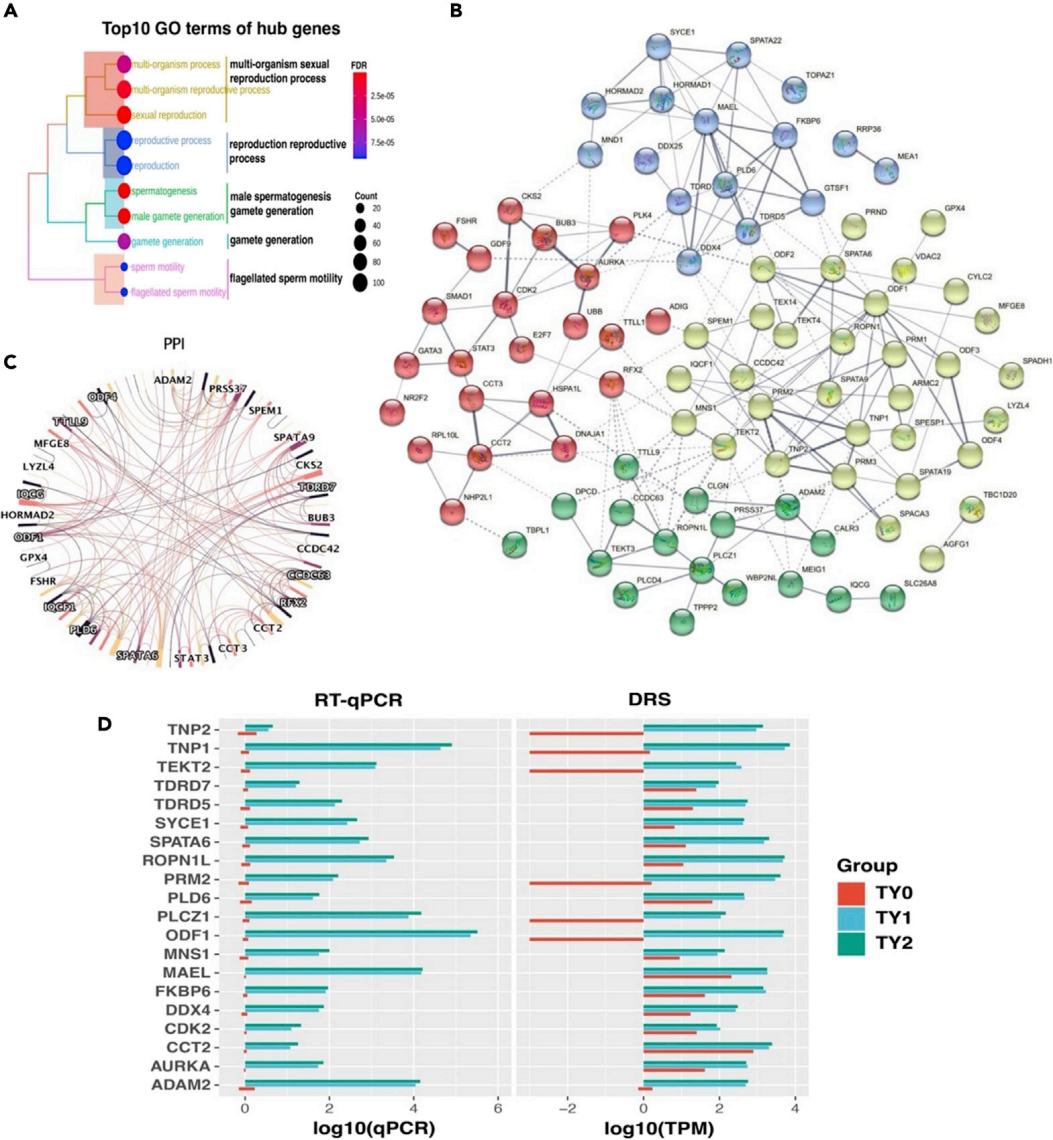
Functional annotation analysis of top20 hub genes (A) GO enrichment analysis of the common hub genes from DEGs and DEIs. (B) Cluster analysis for hub genes from the top10 terms of GO analysis. (C) PPI network of hub genes from the top10 terms of GO analysis. The different color represents the different combined score. (D) RT-qPCR validation of 20 hub genes among the studied groups.

Aiming to examine the impact of differential expression of m6A and m5C sites on the expression level of the hub genes, the expression shift of hub genes related to m6A and m5C sites in the studied groups was verified. We found that the differentially expressed m6A and m5C sites occurred in 2,091 and 1,448 genes, respectively, accounting for 41.46% and 28.71% of total the hub genes (Figure S9). Similarly, they had distinct expression patterns in pairwise comparison groups (Figure 5J). In m6A-modified genes, TY1 and TY2 groups had 1,795 and 1,829 upregulated genes and 931 and 934 downregulated genes, respectively, compared to TY0 group (Figure 5I). Moreover, MeRIP-Seq was used to validate the reliability of m6A-modified hub genes. In total, 67.72% (1,416/2,091) of m6A-modified genes predicted by DRS were also detected by MeRIP-Seq (Figure S8B). Importantly, the hierarchical cluster analysis of these hub genes further revealed that a similar expression trend was observed between DRS and MeRIP-Seq results (Figure S8C). Regarding m5C-modified genes, 387 and 114 upregulated genes and 272 and 147 downregulated genes were detected in TY1 and TY2 groups in comparison with TY0 group, respectively (Figure 5I). Pairwise comparison between TY1 and TY2 groups revealed that TY2 had 54 downregulated and 6 upregulated m6A-modified genes and 303 downregulated and 225 upregulated m5C-modified genes, compared to TY1.

### Function analysis of hub genes related to testis weight

To explore the functional characterization of hub genes related to testis weight, the Gene ontology (GO) analysis was initially employed. The results showed that most of the hub genes were enriched in the sexual reproduction, followed by the male gamete generation, spermatogenesis, multi-organism reproductive process, multi-organism process, gamete generation, reproductive process, reproduction, flagellated sperm motility, and sperm motility (Figure 5A). Subsequently, the 114 genes enriched for the top10 GO terms were used to conduct the protein-protein interaction (PPI) analysis using the STRING database. For them, a total of 87 hub genes were grouped into 3 PPI networks and divided into 4 clusters based on the K-means analysis implemented in the STRING database (Figures 5B and 5C). According to the combined score values, the top20 hub genes related to testicular development and spermatogenesis are listed in Table 3. For them, both cluster 2 and cluster 4 had 7 hub genes, while clusters 1 and 3 had 3 hub genes per each. To validate these findings, qPCR showed a similar tendency of the mRNA expression level among top20 hub genes (Figure 5D). These results indicate that the top20 hub genes might play vital roles in testicular development and spermatogenesis.

**Table 3:** Information on top20 hub genes related to testicular development and spermatogenesis in Simmental bulls

Symbol	Combine score	Cluster	Sexual maturity (before vs. After)				Description
			TY0 vs. TY1	FDR	TY0 vs. TY2	FDR	
ODF1	7.91	Cluster2	14.5543	3.95E-33	14.6989	8.23E-34	Outer Dense Fiber of Sperm Tails 1
MAEL	7.21	Cluster4	2.8705	2.68E-64	2.93819	2.00E-67	Maelstrom Spermatogenic Transposon Silencer
TNP1	7.2	Cluster2	12.8210	5.17E-34	13.3427	8.43E-37	Transition Protein 1
PRM2	6.84	Cluster2	12.0555	3.18E-30	12.5487	1.01E-32	Protamine 2
TNP2	5.86	Cluster2	11.9647	3.73E-22	12.8235	2.27E-25	Transition Protein 2
ROPN1L	5.48	Cluster3	8.4001	7.75E-205	8.5629	2.61E-213	Rhophilin Associated Tail Protein 1 Like
PLD6	5.43	Cluster4	2.6592	7.04E-24	2.5045	3.05E-21	Phospholipase D Family Member 6
AURKA	5.38	Cluster1	3.5837	4.52E-47	3.5455	4.54E-46	Aurora Kinase A
TDRD5	5.36	Cluster4	4.8493	9.80E-42	4.7966	7.62E-41	Tudor Domain Containing 5
CCT2	4.88	Cluster1	1.0416	1.05E-11	1.3234	2.33E-18	Chaperonin Containing TCP1 Subunit 2
TEKT2	4.81	Cluster2	10.5440	8.30E-17	10.4852	1.13E-16	Tektin 2
MNS1	4.73	Cluster2	3.4883	6.60E-11	4.2998	1.95E-16	Meiosis Specific Nuclear Structural 1
DDX4	4.72	Cluster4	3.8703	9.89E-31	3.9995	6.55E-33	DEAD-Box Helicase 4
FKBP6	4.57	Cluster4	5.1335	1.56E-144	4.9299	4.33E-133	FKBP Prolyl Isomerase Family Member 6 (Inactive)
CDK2	4.4	Cluster1	1.9780	5.40E-05	1.8806	0.0001	Cyclin Dependent Kinase 2
PLCZ1	4.38	Cluster3	9.0478	1.70E-12	9.4896	1.05E-13	Phospholipase C Zeta 1
SPATA6	4.33	Cluster2	7.1367	3.37E-110	7.5028	5.22E-122	Spermatogenesis Associated 6
ADAM2	4.16	Cluster3	8.0960	3.14E-31	8.3089	6.55E-33	ADAM Metallopeptidase Domain 2
SYCE1	3.66	Cluster4	5.8507	1.60E-48	6.0265	1.41E-51	Synaptonemal Complex Central Element Protein 1
TDRD7	3.45	Cluster4	1.8349	0.0001	1.9060	7.20E-05	Tudor Domain Containing 7

### MAEL gene contributes to testicular development through post-transcriptional modification and splicing

**Figure 6:**
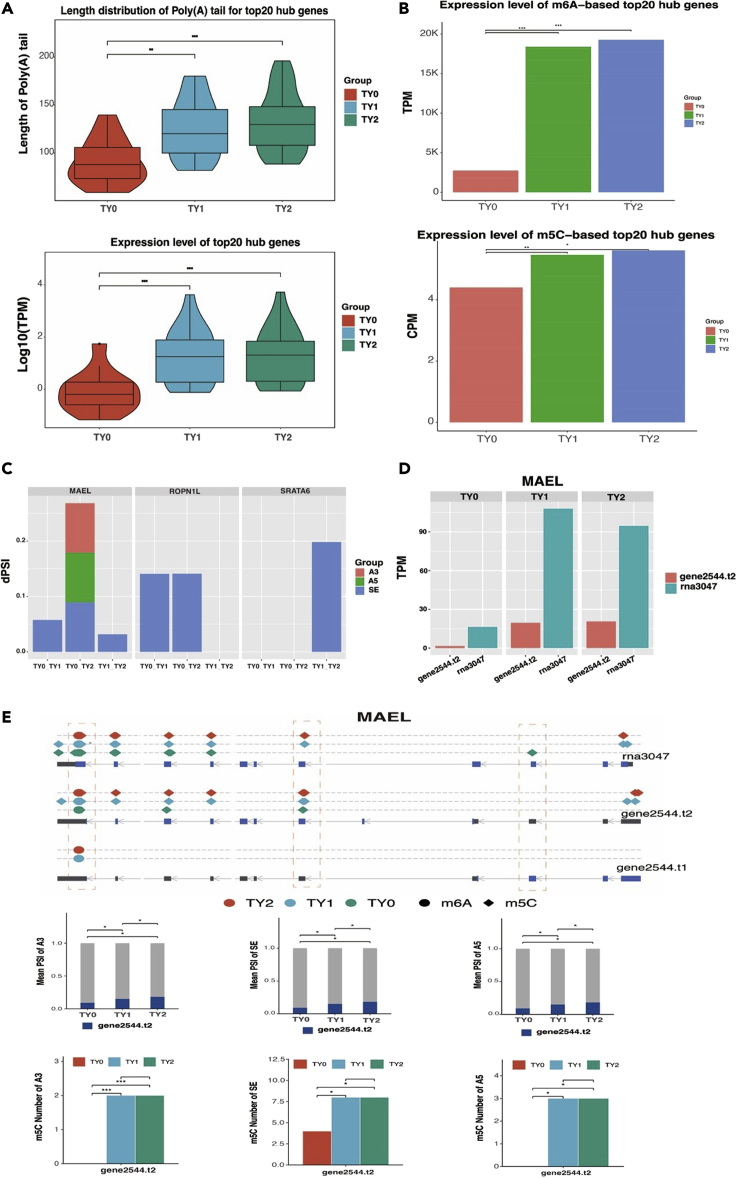
Effect of RNA processing events on the expression level of top20 hub genes (A) Length distribution and expression level of top20 hub genes contribute by poly(A) tail. (B) Expression level of top20 hub genes contribute by m6A and m5C. (C) DAS events of three hub genes. (D) Expression distribution of *MAEL* gene at isoform level. (E) Example locus illustrating the relationship between expression levels and post-transcription regulation in the *MAEL* gene. Top, we only display the m6A and m5C sites which passed the threshold of 0.9, aiming to the visualization clearly; Bottom, all the m5C sites around 100 bp of splicing site were calculated. ∗ indicates significant difference (p < 0.05). ∗∗ indicates significant difference (p < 0.01). ∗∗∗ indicates significant difference (p < 0.001).

In order to further analyze the role of RNA processing events during testicular development and spermatogenesis, we explored the impact of RNA processing events on the expression level of the top20 hub genes. The results showed that the average length of poly(A) tail for the top20 hub genes increased with the increasing of the developmental stage of the testis (Figure 6A). In addition, we found that both m6A and m5C contributed to the expression levels of top20 hub genes before and after sexual maturity (Figure 6B). The results revealed that both length of poly(A) tail and RNA modifications played a vital role in the expression level of hub genes related to testis weight. Nevertheless, we found only three hub genes (*MAEL*, *ROPN1L*, and *SRATA6*) displayed the DAS events across all developmental stages, with *MAEL* showing the highest occurrences (Figure 6C).

Using *MAEL* gene as an example, we further explored the effect of various RNA processing events on gene expression. The results revealed that the *MAEL* isoforms had diverse expression levels at different developmental stages, of which *rna3047* and *gene2544.t2* isoforms were detected (Figure 6D). The *rna3047* isoform had a higher expression level compared to *gene2544.t2* isoform. Interestingly, we also observed that three DAS events including A3, A5, and SE occurred at *gene2544.t2* isoform (Figure 6E, top). More importantly, these DAS events in *gene2544.t2* isoform displayed a significant (p < 0.05) difference among the three stages of testicular development (Figure 6E, mid). These results imply that such DAS events contributed to the expression of *MAEL* gene at the isoform level. Moreover, we found that m5C sites were abundant at the splicing site of the above-mentioned DAS events. In particular, no matter what DAS events were used to make the *gene2544.t2* isoform, the m5C numbers in the TY1 and TY2 groups were significantly (p < 0.05) higher than that in the TY0 group (Figure 6E, bottom). These results point out that the m5C site might increase the occurrence of DAS events, which in turn increase the expression of *MAEL* gene by producing different isoforms.

## Discussion

The present study demonstrated the effectiveness of DRS to characterize RNA molecules at different stages of testicular development. A total of 5,043 differential expression hub genes associated with testicular weight were identified, revealing AS at sexual maturity. Importantly, m6A and m5C modifications impacted the expression of hub genes during testis development. MAEL gene expression at isoform level was significantly affected by m5C-mediated splicing events that promote spermatogenesis.

Testicular development and spermatogenesis involve numerous molecular and cellular events that have been widely studied in model organisms.^40^^,^^41^^,^^42^ Transcriptomics techniques such as mRNA profile,^43^ microRNAs,^44^ long noncoding RNA,^45^ circular RNA,^46^ and m6A profile^39^ have played a vital role in elucidating the molecular mechanisms underlying bovine testicular development and spermatogenesis. It has been known that spermatozoa develop from undifferentiated spermatogonia in mammalian testis through the process of spermatogenesis, involving spermatogonial mitotic proliferation, two successive meiotic divisions, and dramatic morphological changes from haploid spermatids to mature sperm through spermiogenesis (including nuclear condensation, acrosome formation, and assembly of flagellum). Beyond this, somatic cells constitute the testicular microenvironment or niche, which is essential for continuous spermatogenesis.^47^ Nevertheless, the bovine transcriptome is not well characterized yet by long-read RNA sequencing which has the potential to reveal hitherto unknown genes, correct mis-annotations, and expand isoform diversity.^48^ Therefore, the characterization of the bovine testis transcriptome using long-read sequencing is highly required.

In this study, using the PromethION sequencer platform, approximately 50.77 million long reads from 9 bull testis tissues at three different developmental stages were generated. The analysis of these reads demonstrated that the high-quality DRS data obtained are appropriate to be used for further analyses. Subsequently, we successfully assembled the collapsed transcript sequences comprising 97,802 transcripts encoding 56,560 genes, of which 21,427 were previously annotated while 45,072 were novel genes. These DRS data generated such abundant splice variants that accounted for approximately 2.5-fold increase in the isoforms and a 2-fold increase in the genome size of the current bovine genome (*Btau_5.0.1*). Interestingly, our study added about 50,000 novel genes to the current bovine genome, indicating that the bovine transcriptome annotation is far from complete. Notably, the numbers of isoforms and genes in our transcriptome are still higher than those of the latest annotated bovine reference genome (*ARS-UCD1.2*)^49^ since the annotated file of cattle genome was generated by the short-read sequencing. We also provided optimization for 1,861 reference transcripts encoding 1,428 genes in the current NCBI annotation. The huge improvement of annotation by DRS technology is further supported by previous studies.^25^^,^^50^^,^^51^

Identifying candidate genes is an important application of transcriptome analysis. Many studies have demonstrated that short-read sequencing has successfully identified candidate genes related to traits of interest in different livestock species.^52^^,^^53^^,^^54^ On the contrary, studies on the application of long-read sequencing such as DRS technology in farm animals are limited. Several studies have reported the application of DRS in the identification of DEGs and/or DEIs.^27^^,^^55^^,^^56^ In the present study, a total of 15,636 DEGs and 19,321 DEIs were identified between the pairwise comparison groups, of which 9,641 transcripts were shared between DEGs and DEIs. The majority of the DEGs and DEIs occurred between TY0 and TY1/TY2 groups, indicating the presence of large differences in gene expression profiles before and after sexual maturity, which may be related to testicular development and spermatogenesis. Similar results were reported in our previous study conducted on Xia-Nan cattle.^39^ It is noted that the number of biological replicates used in this study has to be taken with caution because it is inferior to that recommended by Schurch et al..^57^ On the other hand, Gleeson et al.^48^ deemed that the number of biological replicates required to identify differential expression may be lower for DRS due to the low level of bias and the possibility of highly accurate quantification. In fact, the most recent application of DRS technology was performed using only two replicates for each sample.^25^^,^^27^^,^^48^ Thus, we have reason to believe that the DEGs and DEIs identified by DRS technology in this study are reliable.

In mammals, testis weight varies with increasing age, implying that it can be set as an important indicator for measuring testicular development and sperm production. One of the valuable contributions of the present study is that the hub genes related to testis weight were further identified by the WGCNA algorithm. In this regard, we observed that both DEGs and DEIs within the turquoise module had a strong positive correlation with the testis weight. For those DEGs and DEIs, Venn’s analysis revealed that 5,043 genes are shared and well defined as hub genes. Importantly, GO analysis demonstrated that most of the hub genes are enriched in the GO terms related to sexual maturity, gamete generation, and spermatogenesis. These results point out that those hub genes play a crucial role in testicular development and spermatogenesis. Although the current study was conducted on limited samples, the obtained results are similar to that of previous studies with larger sample sizes.^35^^,^^58^^,^^59^ Later, qPCR and other analyses were performed to verify the preliminary results.

One contribution of DRS technology is its ability to detect poly(A) tail length. Evidence showed that the poly(A) tail length has fundamental importance to determine gene activity.^29^^,^^60^^,^^61^ In the present study, we further explored the relationship between poly(A) tail length and the expression level of hub genes. The obtained results revealed that the poly(A) tail length of hub genes derived from TY0 was higher in count than that of the reached-maturity groups of TY1 and TY2. Meanwhile, we observed that the poly(A) tail length had negative correlations with the TPM values of hub genes among all groups. These results indicate that the inverse relationship between poly(A) tail length and expression level may vary depending on the testis developmental stages. Similar results were reported in other studies.^25^^,^^27^^,^^29^ In short, our findings reveal that the length of the poly(A) tail could regulate the stability of hub gene expression, thereby influencing the testis weight.

The analysis of AS events contributes to a better understanding of gene functions in the developmental process of animal species.^28^^,^^62^^,^^63^ In the current study, the most abundant splicing events within the hub genes were the SE type, accounting for 23.63% of total AS events. Chacko and Ranganathan^64^ also found that the majority of SE events were present in the bovine genome. In addition, previous studies have demonstrated that the SE events are accumulated in the testis,^21^^,^^65^^,^^66^ which further supports the reliability of our results. A total of 2,795 DAS events were detected between the pairwise comparison groups, of which 655 were of A3 type, followed by A5 type (653) and SE type (552). This indicates that A3 events might play a key role in regulating the transcript diversity of hub genes. A significant expression difference for DAS events between TY0 and TY1/TY2 groups was observed, but this scenario was not found between TY1 and TY2 groups. Such DAS events only occurred in 716 genes, accounting for 14.20% of hub genes. These results show that the hub genes related to AS events could regulate the expression level, hence influencing the testis weight.

RNA modification is known to have several types (over 160) that play key roles in regulating the fate of RNA.^67^ Evidence showed that both m6A and m5C are the most abundant mRNA modifications that had multiple key regulated roles in RNA processing, including RNA stability, translation, AS, and nuclear export. Thus, identifying m6A and m5C profiles is of great importance to discover the potential impact of hub genes on both testis development and spermatogenesis. In the present study, we observed that a total of 32,716 m6A and 209,597 m5C sites occurred in 2,091 and 1,448 hub genes, respectively, accounting for 41.46% and 28.71% of the total hub genes. These methylated sites were enriched near the stop codon and 3′UTR regions. The pattern detected in this study is similar to previous reports,^27^^,^^31^^,^^68^ which further validates the reliability of our results. Likewise, these methylated genes had distinct expression patterns of m6A and m5C between pairwise comparison groups. We observed that 448 hub genes had both m6A and m5C events. Importantly, most of these hub genes were enriched into the top3 GO terms: cytoplasm, cytosol, and nucleus of CC; translation, spermatogenesis, and vesicle-mediated transport of biological processes (BP); and structural constituent of ribosome, RNA binding, and ATP binding of molecular functions (MF). These results indicate that the hub genes involved in the basic biological functions of sperm cells and their development are more closely related to the simultaneous multiple methylations.

Spermatogenesis starts at puberty and is the process of spermatozoa formation that consists of spermatogonia mitosis, spermatocyte development and meiosis, and spermatids elongation. To further explore the role of hub genes in spermatogenesis, we sought to verify a potential PPI relationship between the hub genes within top10 GO terms. The results revealed that a total of 87 hub genes are grouped into 3 PPI networks and divided into 4 clusters based on the K-means analysis. We also focused on the top20 hub genes related to testicular development and spermatogenesis according to the combined score values. Importantly, some hub genes were associated with testicular development and spermatogenesis, such as *ODF1*,^69^ *PRM2*,^70^ and *ROPN1L*^71^ for the sperm motility; *MAEL*,^72^ TNP1/TNP2,^73^ *PLD6*,^74^ *TDRD5*,^75^ *TEKT2*,^76^ *MNS1*,^77^ *SPATA6*,^78^ and *ADAM2*^79^ for spermatogenesis; *DDX4*^80^ and *TDRD7*^81^ for germ cell development; *FKBP6*^18^ for male fertility and homologous chromosome pairing in meiosis; and *PLCZ1*^82^ for monospermic fertilization. In addition, four genes (*SYCE1*, *CDK2*, *CCT2*, and *AURKA*) were reported to be associated with mitosis and meiosis divisions,^83^^,^^84^^,^^85^^,^^86^ which are the basic biological process of testicular development and spermatogenesis.^87^ Moreover, our results demonstrate that RNA modification and poly(A) tail contributed to the expression levels of most top20 hub genes before and after sexual maturity. Despite this finding, only three hub genes (*MAEL*, *ROPN1L*, and *SRATA6*) displayed the DAS events among the testis developmental stages, with *MAEL* gene showing the most occurrences. Evidence showed that *MAEL* gene was localized in both pachytene spermatocytes and chromatid bodies of spermatids in rats,^17^ implying that it potentially plays a vital role in spermatogenesis. Furthermore, we observed that the A3, SE, and A5 events were detected in the *gene2544.t2* isoforms and increased isoform expression both before and after sexual maturity. Many studies have demonstrated that AS is indispensable to increase the complexity of gene expression, which plays a key role in various biological processes.^37^^,^^88^^,^^89^ This means that these splicing events play a dominant role in the complexity of *MAEL* gene expression. Notably, we found that m5C sites are the most abundant at the splicing site of SE in *gene2544.t2* isoform, followed by A5 and A3 events. Notably, we observed that the three DAS events in TY1/TY2 groups were significantly higher than those in TY0 group, indicating that these m5C sites might increase the occurrence of DAS events. Interestingly, evidence showed that one m5C site (m5C_hg19_71877) within the splicing site of *MAEL* gene contributed to its expression in human testis tissue,^90^ which is similar to our results for *MAEL* gene in cattle. These results point out that m5C site might play a vital role in the occurrence of splicing events. Further experimental validation is needed to find the major sources of variation and control regarding the splicing events.

In summary, we investigated the complexity and dynamics of the bull testis transcriptome at different developmental stages using DRS technology. We constructed and characterized the bovine testis transcriptome and further optimized the current bovine transcriptome annotation. Furthermore, some hub genes related to testicular development and spermatogenesis were identified. The obtained results confirm that the poly(A) tail length, alternative splicing, and RNA modification have different functional effects on the expression level of hub genes, thereby influencing the testis weight. Notably, m5C-mediated splicing events were found to contribute to the expression of *MAEL* gene. This study strongly highlights how RNA processing events regulate distinct expression of hub genes related to testicular development and spermatogenesis. The identified hub genes in the present study will play vital roles in dissecting the mechanisms of testicular development and spermatogenesis.

### Limitations of the study

In this work, we characterized the dynamics and complexity of the bovine testis transcriptome during different developmental stages. We identified some hub genes related to testis weight, and their transcript expression levels were affected by the poly(A) tail length, alternative splicing, and RNA modifications. Importantly, we found that m5C-mediated splicing events significantly increased *MAEL* gene expression at the isoform level, likely promoting spermatogenesis. Nevertheless, limited information on direct evidence of m5C modification function for *MAEL* gene expression, assessment of progeny outcomes from tested bulls, and cell composition of testicular tissue used for DRS sequencing are the limitations of the study.

## STAR★Methods

### Key resources table

**Table:** 

REAGENT or RESOURCE	SOURCE	IDENTIFIER
**Biological samples**		

Simmental bull’s testes	This paper	N/A

**Critical commercial assays**		

Total RNA Kit I (For DRS)	Omega Bio-tek (USA)	Cat#:R6834
NEBNext Poly(A) mRNA Magnetic Isolation Module	New England Biolabs (USA)	Cat#:E7490S
Super-Script III Reverse Transcriptase,	Thermo Fisher Scientific (USA)	Cat#:18080044
dNTP solution New England Biolabs (USA)	New England Biolabs (USA)	Cat#:NEB N0447

**Deposited data**		

Direct RNA sequencing data	NCBI SRA database	BioProject: PRJNA831889
Raw Illumina sequencing data	NCBI SRA database	BioProject: PRJNA832033

**Experimental models: Organisms/strains**		

Simmental bulls	Henan Dingyuan Cattle Breeding Co., Ltd	N/A

**Software and algorithms**		

Guppy	kahlke, T^91^	ver.5.0.16
Nanofilt	De Coster et al.^92^	ver.2.7.1
fmlrc2	Wang et al.^93^	ver. 0.1.4
minimap2	Li^94^	ver. 2.17-r941
Samtools	Danecek et al.^95^	ver.1.11
FLAIR	Tang et al.^96^	ver.1.4
Gffcompare	Pertea et al.^97^	ver.12.1
DESeq2	Love et al.^98^	ver. 1.26
stringtie2	Kovaka et al.^99^	ver.1.3.6
WGCNA	Langfelder et al.^100^	ver.1.70–3
clusterProfiler	Wu et al.^101^	ver.4.6.0
Nanopolish	De Coster et al.^92^	ver. 0.13.2
SUPPA2	Trincado et al.^102^	ver.2.3
Tombo	Stoiber et al.^103^	ver.1.5
MINES	Lorenz et al.^104^	ver.1.5
methylKit	Akalin et al.^105^	ver.0.99.2
KOBAS-i	Bu et al.^106^	ver.3.0
TrimGalore	FelixKrueger et al.^107^	ver. 0.6.7
HISAT2	Kim et al.^108^	ver.2.1.0
exomePeak2	Meng et al.^109^	ver.1.10.0

### Resource availability

#### Lead contact

Further information and requests for resources and reagents should be directed to and will be fulfilled by the lead contact, Tingxian Deng (dtx282000@163.com).

#### Materials availability

This study did not generate new materials.

### Experimental model and subject details

#### Ethical approval

All procedures and protocols performed in this study were carried out under the Ethics of Henan Agricultural University Animal Care and Use Committee (approval number: HENAU-2021-023).

#### Animal sample collection

A total of 9 clinically healthy Simmental bulls maintained at Henan Dingyuan Cattle Breeding Co. Ltd (Zhengzhou, China) were selected according to their developmental stages and physiological status. These animals were divided into three groups: 1) prepuberty group (TY0) consisted of three calves aged one week, 2) puberty group (TY1) contained three bulls at about 1-year-old producing at least 50 × 10^6^ sperm per ejaculate with >10% motility rate,^110^ and 3) post-puberty group (TY2) comprised of three bulls with approximately 2-year-old showing sexual maturity signs. All young bulls were reared in individual pens indoors. Roughage, concentration supplements and clean water were available *ad libitum*. The calves were fed colostrum. Immature bulls forming the prepubertal group were isolated from the herd at approximately one week after birth. Within one month after forming the puberty and post-puberty experimental groups, semen samples were collected using an artificial vagina and were used for further analysis. The selected bulls for this study were given general anesthesia and then their left testes were collected posterior to castration procedure by professional veterinarians, as described by Sakase et al..^111^ Two samples per each animal were collected from the testicular lobule. The first portion was preserved in 4% formaldehyde solution for the histological assessment, while the second portion was immediately stored in liquid nitrogen until performing RNA extraction.

### Method details

#### Semen phenotype analysis and testicular morphology

To investigate the differences in the sperm production ability of bulls between puberty and post-puberty groups, we evaluated bull semen quality within only one month in each group. Sperm concentration was assessed using the Sperm Densitometer (Minitube SDM6, Germany), while sperm motility was evaluated based on the method described by Björndahl et al..^112^ Statistical differences between the puberty and post-puberty groups regarding the semen quality parameters were determined by the *t*-test implemented in the R package. To compare the morphological differences of left testes at different development stages, we measured testis length, width, and weight after removal of the tunica vaginalis and epididymis. The length and width of each testis were measured by calipers. The one-way analysis of variance (ANOVA) was conducted to test the presence of significant differences between the contrasting groups using GraphPad Prism 9 software (GraphPad Prism Software Inc., San Diego, USA). A p value of <0.05 was defined as the threshold level.

#### RNA isolation and sequencing

Total RNA was extracted in accordance with the standard protocol of the Total RNA Kit I (Omega Bio-tek, R6834). RNA was cleaned and concentrated by the NEBNext Poly(A) mRNA Magnetic Isolation Module (NEB, E7490S) according to the manufacturer’s instructions. Then, 1 μg of total RNA was extracted from each sample for measuring RNA integrity and concentration using Nanodrop (Thermo Fisher Scientific, Waltham, MA, USA) and Qubit 3.0 Fluorometer (Life Technologies, Carlsbad, CA, USA), respectively.

For the Illumina sequencing, all libraries from purified mRNA were made using the NEBNextUltra™ RNA Library Prep Kit for Illumina (NEB, USA). The quantity and quality of the constructed libraries were evaluated using the Qubit 3.0 Fluorometer (Life Technologies, Carlsbad, CA, USA) and Agilent 2100 bioanalyzer (Agilent Technologies, Santa Clara, CA, USA). The paired-end sequencing with a read length of 150 bp was performed on an Illumina NovaSeq 6000 System. The Illumina sequence data were used later for correcting DRS data.

Nanopore libraries for each sample were generated using the SQK-RNA002 kit (Oxford Nanopore Technologies, Oxford, UK) according to the manufacturer’s instructions. Briefly, mRNA for each sample was first isolated from approximately 50 μg total RNA using the Dynabeads mRNA purification kit (Thermo Fisher Scientific, Waltham, MA, USA). Next, for reverse connector connection, 9 μL prepared RNA, 3 μL NEBNext Quick Ligation Reaction Buffer (NEB, USA), 1 μL RT Adapter (RTA)(SQK-RNA002), and 2 μL T4 DNA Ligase (NEB, USA) were mixed and incubated under 25°C for 10 min. Afterward, 8 μL 5x first-strand buffer (NEB, USA), 2 μL 10 mM dNTPs (NEB, USA), 9 μL Nuclease-free water, 4 μL 0.1 M DTT (Thermo Fisher Scientific, R0861), and 2 μL Super-Script III Reverse Transcriptase (Thermo Fisher Scientific, 18080044) were added to the above-mentioned reaction system of 15 μL, and incubated under 50°C for 50 min, followed by 70°C for 10 min. Reverse-transcribed mRNA was purified with 1.8x AgencourtRNAClean XP beads and washed with 23 μL Nuclease-free water, and subsequently the sequencing adapters were added using 8 μL NEBNext Quick Ligation Reaction Buffer, 6 μL RNA Adapter (RMX), and 3 μL T4 DNA Ligase. The mix was purified and washed again as described above, and then 75 μL RRB (SQK-RNA002) plus 35 μL Nuclease-free water were added. Finally, the reaction system was loaded onto the R9.4 flow cell, and sequencing was performed in the PromethION sequencer (Oxford Nanopore Technologies, Oxford, UK) for 48 h. Three biological replicates were applied for each group.

#### DRS data analysis

Base-calling for reads was performed by the Guppy ver.5.0.16 software^91^ using default RNA parameters. Only “pass” reads (quality score ≥7), as designated by the Nanofilt ver.2.7.1 tool,^92^ were used for subsequent analyses. To improve the quality of ONT data, error correction of the filtered ONT reads was further performed with the Illumina RNA-seq data by using the fmlrc2 ver. 0.1.4 software.^93^ The corrected ONT reads were aligned to the bovine genome (***Btau_5.0.1***) and transcriptomes (***Btau_5.0.1***) using minimap2 ver2.17-r941 software^94^ with parameters ‘-ax splice -uf -k 14’ and ‘-ax map-ont -N100’, respectively. The resulting SAM files were sorted and indexed with samtools ver.1.11 software.^95^ In addition, identification of *de novo* transcript for each DRS dataset was performed using the FLAIR ver.1.4 ^103^ and stringtie2 software,^99^ as described by Soneson et al..^51^ Transcripts identified from each DRS dataset were compared with the annotated reference transcripts using gffcompare ver.12.1 software^97^ with parameters: -R-C-K-M. The gffcompare class codes “i”, “j”, “o”, “u”, and “x” were considered novel transcriptional loci. Salmon ver1.8.0 software^113^ was used to conduct the gene- and isoform-level quantification analysis.

#### Differential gene expression and differential isoform expression analysis

Analyses of differential expression genes (**DEGs**) or differential expression isoforms (**DEIs**) between pairwise comparison groups were performed by the DESeq2 ver1.26 ^107^. Count matrices for gene and isoform levels were generated by a python script (preDE.py) implemented in stringtie2 software.^99^ Only genes and isoforms that had more than one read per million mapped reads in at least two libraries were retained and normalized by DESeq2 ver1.26 ^107^. The false discovery rate (FDR) < 0.05 and fold change >2 were defined as the cutoff criteria for the DEGs and DEIs.

#### Weighted gene co-expression network analysis of DEGs and DEIs

The weighted gene co-expression network analysis (**WGCNA**) method was applied to investigate the DEGs and DEIs affecting both testicular morphology and semen phenotypic traits. Overall, TPM matrix of DEGs and DEIs was first used to predict the power parameter. The suitable soft threshold of 26 and 18 were selected for DEGs and DEI datasets, respectively (Figure S10), as they met the scale-free topology criterion (degree of independence of 0.85). Subsequently, the module was constructed using the one-step method with the dynamic hierarchical tree-cut algorithm. The main parameters were included: minModuleSize = 30, mergeCutHeight = 0.25, maxBlockSize = 6,000 (DEGs) or 4,000 (DEIs), and networkType = “unsigned”. To further analyze the correlation between module and traits of interest, the module eigengenes (**MEs**) were calculated for each module and used to determine their correlation with the traits of interest. After determining the modules of interest, the hub genes within modules were identified. Three parameters (**GS**: the correlation between gene expression and each trait; **MM**: the association between gene expression and each module eigengene; and p value) were employed to mine the hub genes. For the DEG and DEI modules, the hub genes associated with traits of interest were chosen based on the following thresholds: GS > 0.8, MM > 0.8, and p value <0.05. Finally, the common hub genes both in DEGs and DEIs datasets were considered as the “real” hub genes for further analysis. All the above analyses were carried out and implemented in the WGCNA R-package.^100^ The Gene Ontology (**GO**) and Kyoto Encyclopedia of Genes and Genomes (**KEGG**) pathway enrichment analyses of hub genes were performed by the clusterProfiler R package.^101^ The False Discovery Rate (FDR) adjusted p value of <0.05 was set as the significance threshold, and the enrichment results of GO and KEGG pathways in each module of interest were obtained.

#### PolyA tail length analysis of hub genes within target module

To investigate the poly (A) tail length affecting the expression level of hub genes, estimation of the poly (A) tail lengths was performed using the nanopolish ver. 0.13.2 software.^92^ Diff_Median is the difference between the median length of Poly(A) in two groups, which can be used to measure the size and direction of the difference. The difference in poly(A) tail length in hub genes between the pairwise comparison groups was determined using the Mann-Whitney U test. The p values were adjusted for multiple testing using Benjamini-Hochberg correction. The adjusted p value of <0.05 was defined as a significance different threshold.

#### Alternative splicing analysis of hub genes within target module

To explore the effects of alternative splicing (**AS**) on the hub gene expression, the AS model for each sample was predicted using SUPPA2 software,^102^ with the default parameters. Using transcript quantification, SUPPA2 could calculate the inclusion values (PSI) of AS events across multiple samples. The expression of transcript in the TPM units were quantified using Salmon ver1.8.0 software. The differential expressed AS (**DAS**) events of hub genes between the pairwise comparison groups were performed by the DiffSplice command line implemented in SUPPA2 software.^102^ The DAS events for each sample were obtained by selecting AS events with |ΔPSI |> 0.1 and p value <0.05.

#### RNA modification of hub genes within target module

Tombo ver.1.5 software^103^ and MINES^104^ software were utilized for identifying the m5C and m6A modifications of hub genes. Score on each site indicated the fraction of a possible modification on a given site. For the m6A modification, the differential m6A modification [fold changes (FC) ≥ 2 and adjusted p value <0.05] between the pairwise comparison groups were analyzed using the DESeq2 package in R.^98^ Regarding the m5C modification, only the sites with >10 reads covered were selected for further analysis. The logistic regression algorithm implemented in methylKit in R^105^ was used to determine the differentially expressed m5C sites between the pairwise comparison groups. The raw p value was adjusted to the q-value using the sliding linear model (SLIM) method.^114^ The q-value <0.01 and the percent methylation difference larger than 25% were defined as the threshold levels for identifying differential m5C sites. In addition, the different numbers of m5C sites per gene were set as an expression value and used for further analysis. Venn analysis was applied to detect the common genes between m5C and m6A modifications related to genes. Finally, KOBAS-i software^106^ was used to conduct the GO and KEGG enrichment analyses of common genes based on the adjusted p value of <0.05 as a threshold level.

#### Integrated analysis of RNA modification and alternative splicing for hub gene

To investigate the relationship between RNA modification and alternative splicing, we performed the integrated analysis of the two RNA processing events in the *MAEL* gene. In briefly, transcriptomic coordinates of the MAEL gene were first converted into genome positions. RNA modifications including m6A and m5C sites were scanned according to genome position and visualized using the methylation scores greater than 0.9. Likewise, alternative splicing events were displayed according to genome coordinates at isoform level. The t-test was carried out to analyze the difference in significance between alternative splicing events between different developmental stages using the PSI value for MEAL isoforms. Further, the t-test was used to determine the significance of the differences between different developmental stages based on the number of m5C sites located within the 100bp of splicing site. All plots were performed using the ggplot2 package in R.

#### Validation of m6A related to hub genes using MeRIP-Seq

To validate the predicted m6A sites based on Nanopore DRS, we performed the MeRIP-Seq analysis using our previously published bovine testis transcriptome data that was deposited in the NCBI SRA database under the accession number PRJNA776655. The data consisted of 9 samples representing three developmental stages of the bull testicles. Clean reads were generated from the raw data by TrimGalore v0.6.7 software.^107^ Subsequently, the high-quality clean reads were mapped to the bovine reference genome (***Btau_5.0.1***) by HISAT2 ver.2.1.0 software,^108^ with default parameters. Peak calling for all libraries was performed using the exomePeak2^109^ package in R. Also, the exomePeak2 package^109^ was used for the analysis of differential m6A peaks (FC ≥ 2 and adjusted p value <0.05) between the pairwise comparison groups.

#### Quantitative real-Time PCR confirmation

Thirty-one genes or transcripts were selected and analyzed by qRT-PCR. Primers were designed using Primer 5.0 software and synthesized by Sangon Biotech (Shanghai) Co. Ltd (Table S1) The cDNA synthesis was performed by using the RevertAid First Strand cDNA Synthesis Kit (Thermo Fisher Scientific, Waltham, MA, USA), following the manufacturer’s protocols. Subsequently, qRT-PCR was conducted using QuantiNova SYBR Green PCR Kit (QIAGEN, Shanghai, China). The *GAPDH* gene was used for normalizing the relative abundances of genes. The 2^−ΔΔCt^ method^115^ was applied to analyze the data for all samples in triplicate technical replicates.

### Quantification and statistical analysis

The analyzed data were expressed as mean ± SEM. The one-way ANONA analysis was used to determine the statistical significance. Each test was conducted in triplicate. Data analysis was performed by GraphPad Prism 9.0 software (GraphPad Prism Software Inc., San Diego, USA).

## Data Availability

Data availability• The raw Illumina sequencing data for all 9 libraries were deposited in the NCBI Sequence Read Archive (SRA) under accession No. PRJNA832033. The DRS sequencing data for all 9 libraries have been deposited in the NCBI SRA database under the accession number PRJNA831889. Code availability• This paper does not report original code. Additional information• Any additional information required to reanalyze the data reported in this paper is available from the lead contact upon request.
