# Supplementary material for: MAEL gene contributes to bovine testicular development through the m5C-mediated splicing
Source: iScience. 2023 Jan 6;26(2):105941. doi: 10.1016/j.isci.2023.105941 (PMC9876746; doi:10.1016/j.isci.2023.105941)
Supplement: Document S1. Figures S1–S10 [file mmc1.pdf]

iScience, Volume *26*

Supplemental information

MAEL gene contributes to bovine testicular development through the m5C-mediated splicing

Shenhe Liu, Xiaoya Ma, Zichen Wang, Feng Lin, Ming Li, Yali Li, Liu Yang, Hossam E. Rushdi, Hasan Riaz, Tengyun Gao, Liguo Yang, Tong Fu, and Tingxian Deng

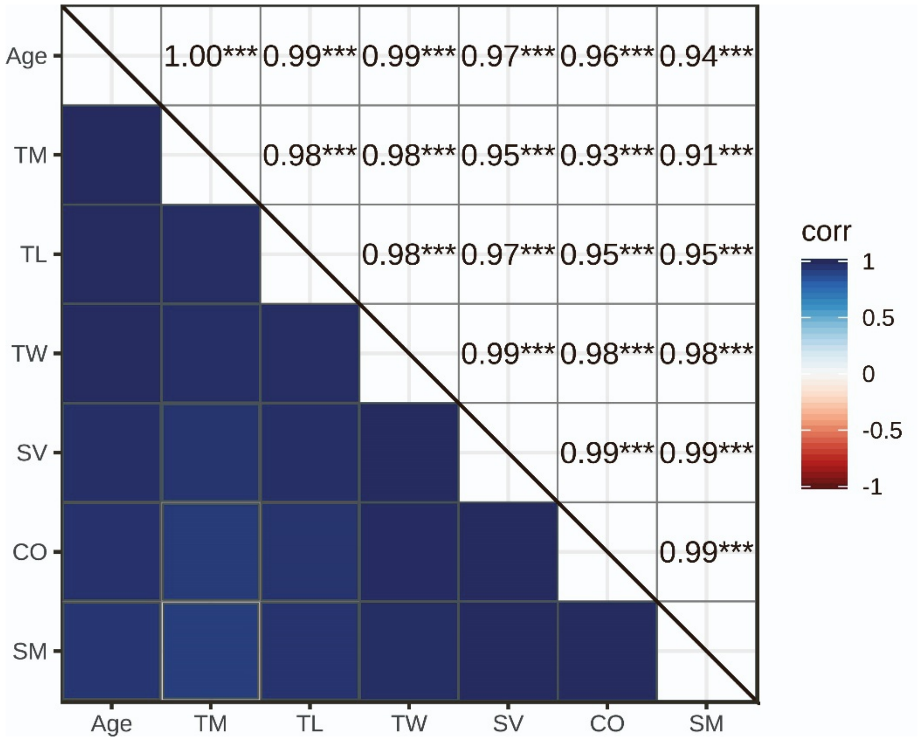

Figure S1. Correlation among different morphological features and semen quality parameters in bulls, Related to Table 1. The asterisk indicates the significant levels.

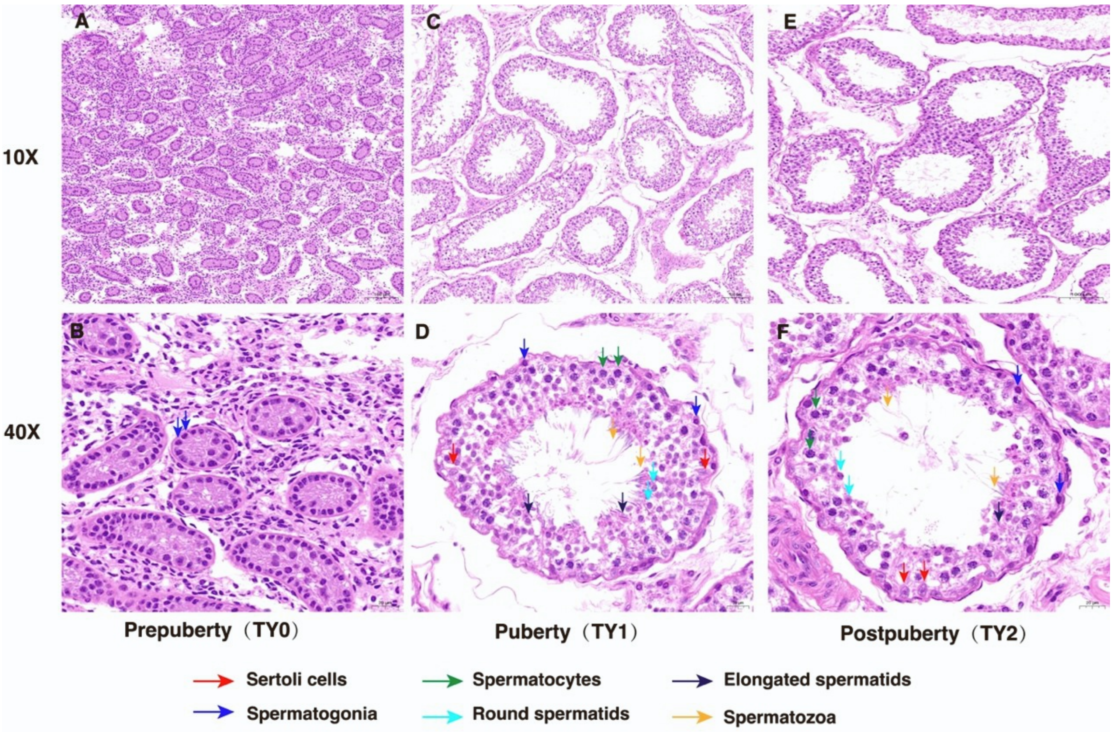

Figure S2. Histomorphology analysis of bull testicular tissues at three developmental stages was performed under a microscope at the magnifications of 10X and 40X, Related to Table 1. Sections (A) and (B) characterize the morphology of prepuberty testes, sections (C) and (D) represent the morphology of puberty testes, as well as sections (E) and (F) signify the morphology of post-puberty testes. The different color arrows exhibited different germ cell types.

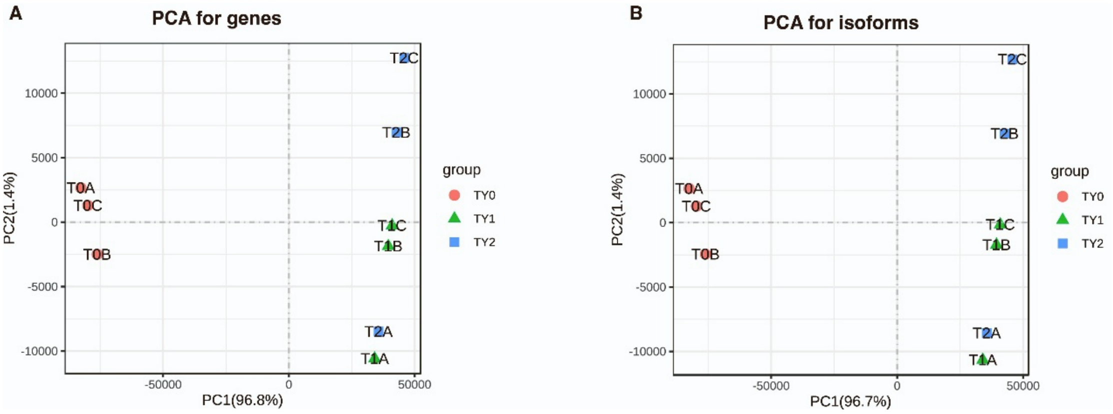

Figure S3. PCA analysis of genes and isoforms among the studied groups, Related to Figure 3. The different colors and shapes indicate the different groups.

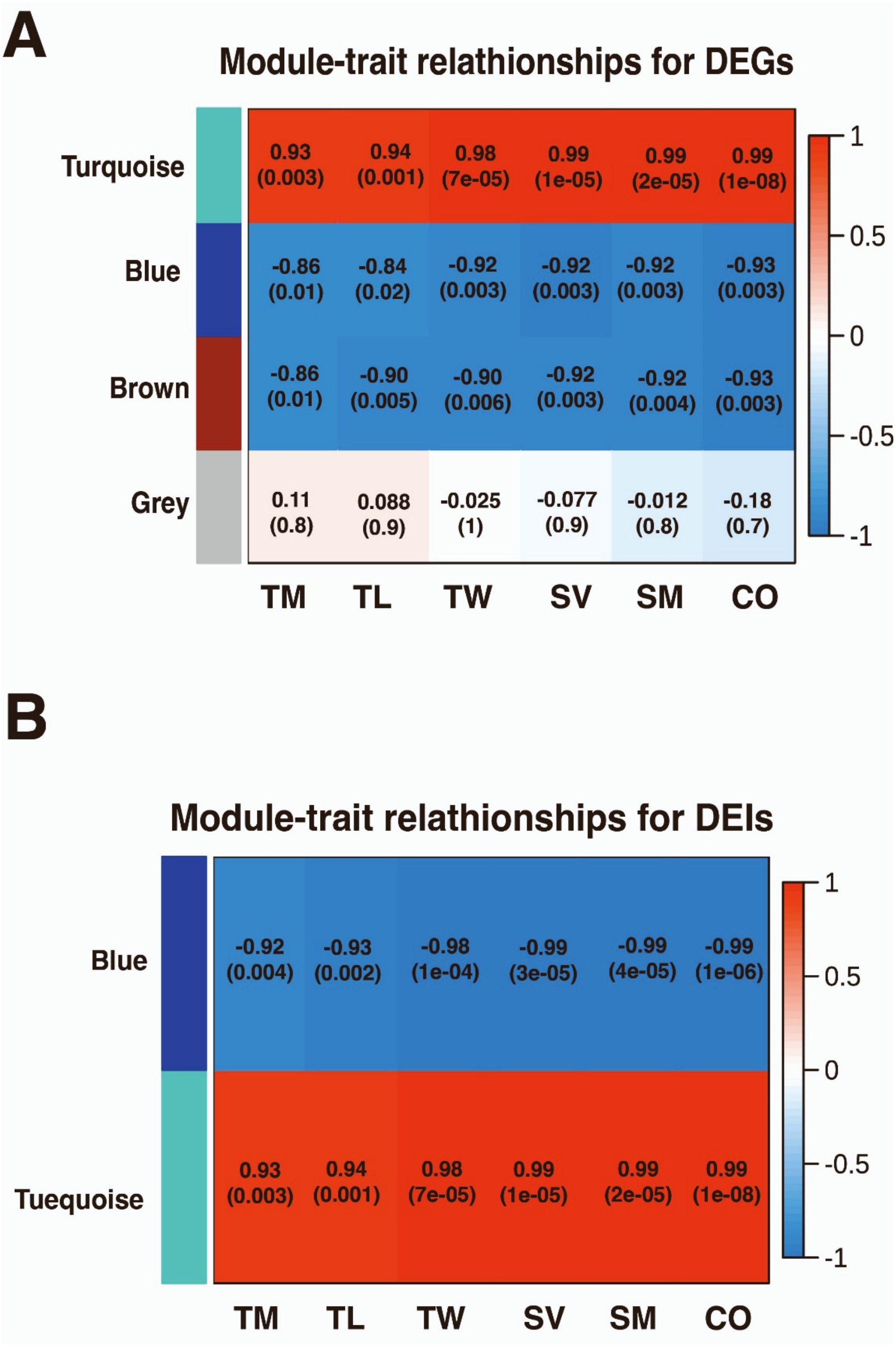

Figure S4. Relationship between traits and modules based on WGCNA analysis, Related to Figure 4.

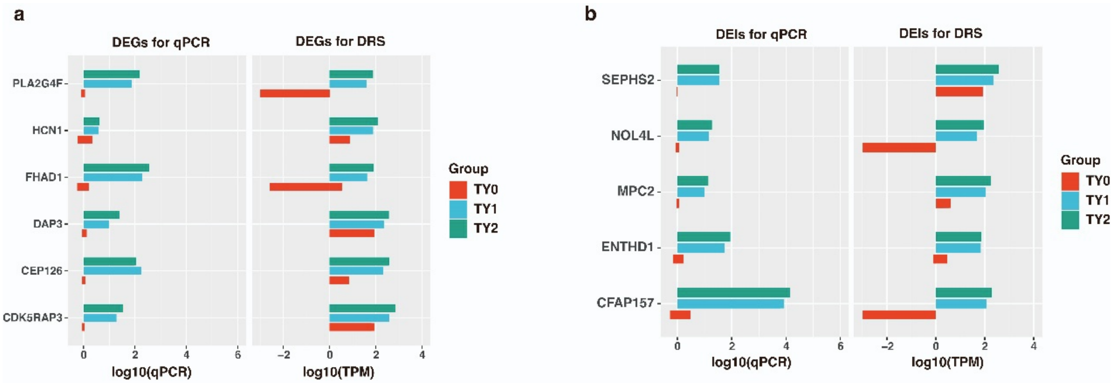

Figure S5. qRT-PCR validated the reliability of 11 randomly selected hub DEGs and DEIs, Related to Figure 4.

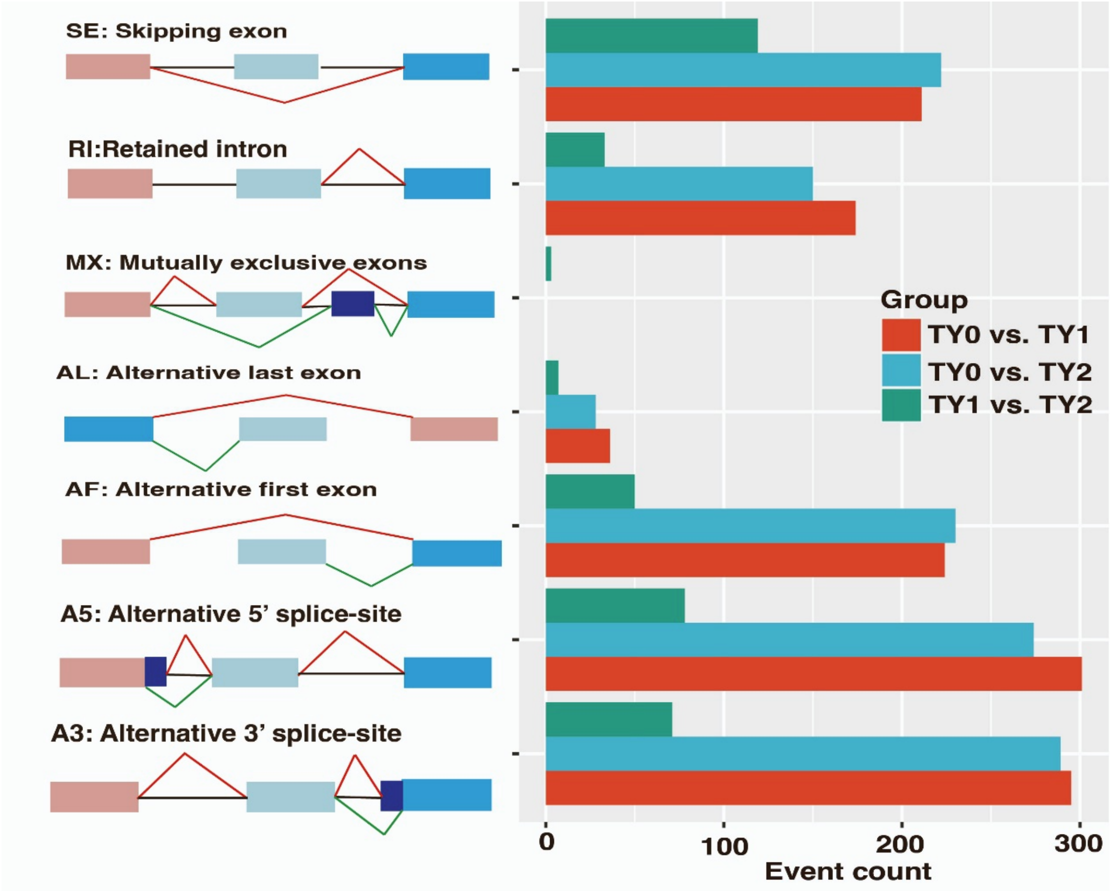

Figure S6. Differential alternative splicing analysis for hub genes among different developmental stages, Related to Figure 4.

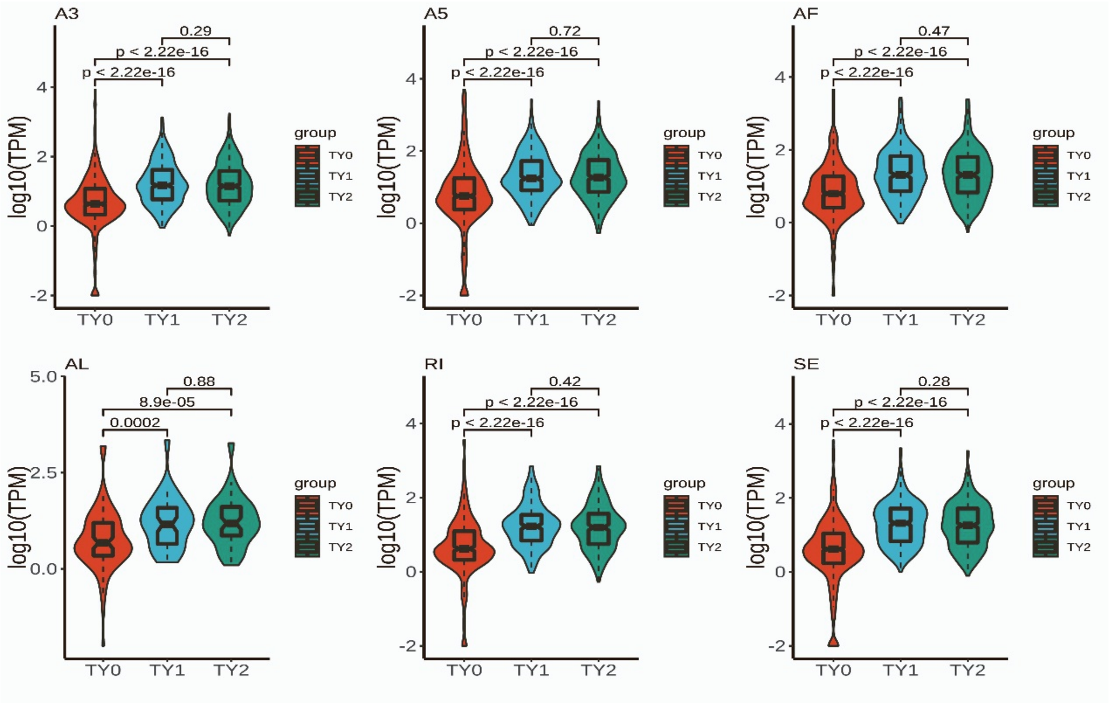

Figure S7. Expression analysis for different DAS events among different developmental stages, Related to Figure 4.

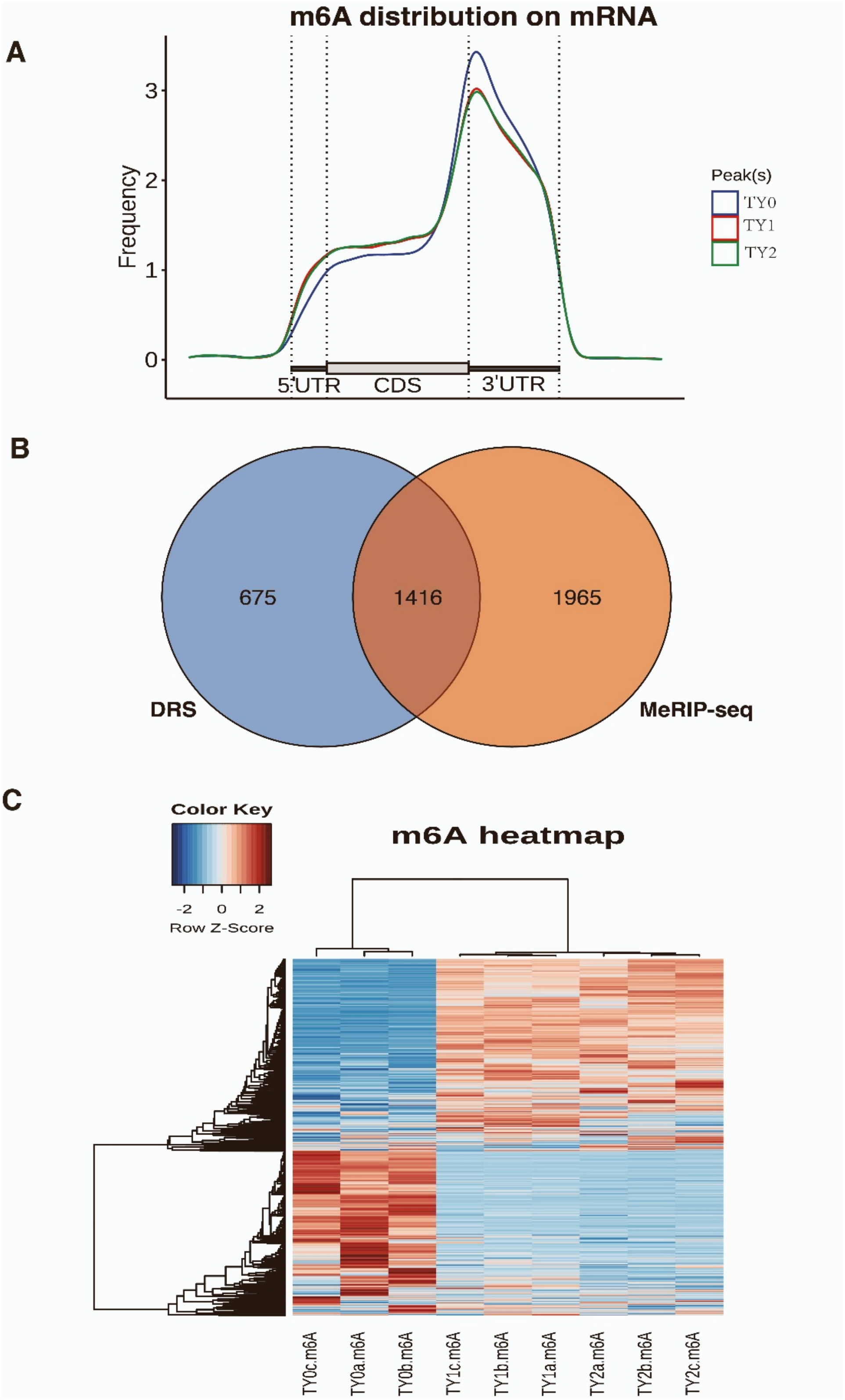

Figure S8. MeRIP-Seq validated the m6A modifications for hub genes based on DRS data, Related to Figure 4.

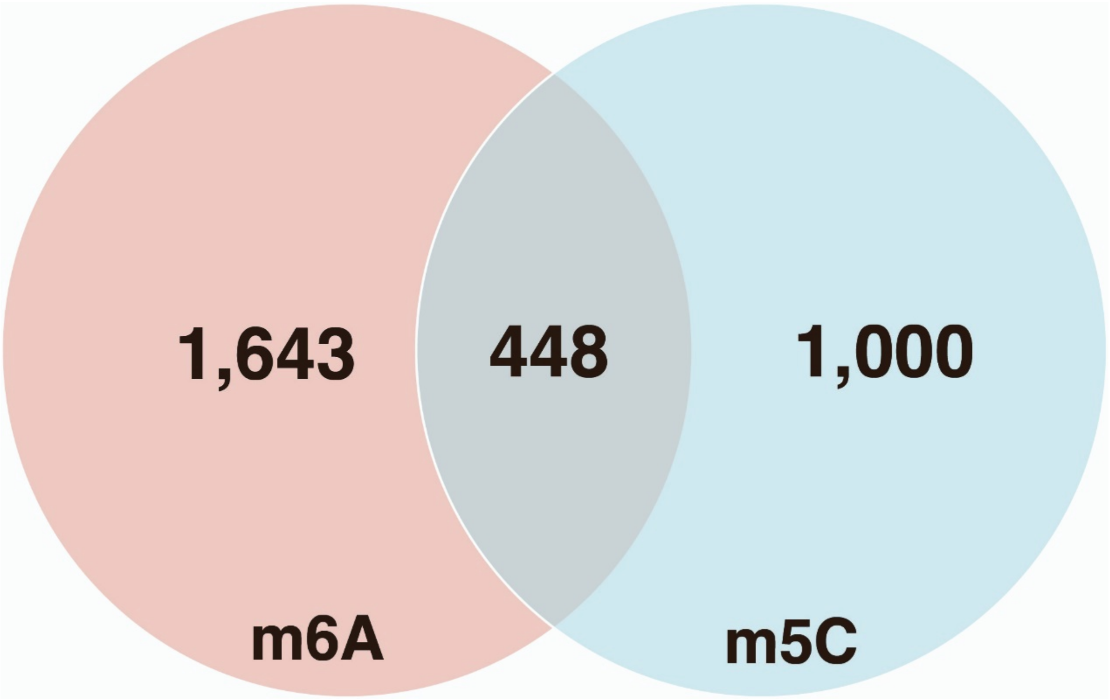

Figure S9. Venn analysis for m6A and m5C sites, Related to Figure 5.

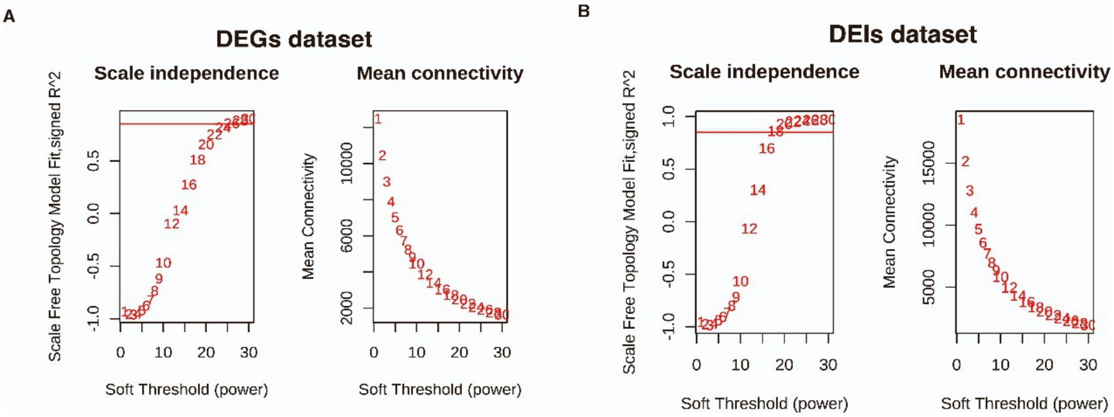

Figure S10. Identification of beta soft threshold values for the DEGs and DEIs datasets, Related to Figure 6.
